# Beyond Clinical Trials: Experience with Bimekizumab in 3 Challenging Cases of Psoriatic Arthritis

**DOI:** 10.31138/mjr.071125.est

**Published:** 2026-01-08

**Authors:** Evangelos Papadimitriou, Ioanna Katsigianni, Marina Papoutsaki, Michail Visvikis, Theodoros Dimitroulas, Christos Koutsianas, Charalampos Papagoras

**Affiliations:** 1 First Department of Internal Medicine, University Hospital of Alexandroupolis, Democritus University of Thrace, Alexandroupolis, Greece;; 2 Fourth Department of Internal Medicine, Hippokration Hospital, Aristotle University of Thessaloniki, Thessaloniki, Greece;; 3 First Department of Dermatology-Venereology, Faculty of Medicine, “Andreas Sygros” Hospital for Skin and Venereal Diseases, National and Kapodistrian University of Athens, Athens, Greece;; 4 Joint Academic Rheumatology Program, Clinical Immunology-Rheumatology Unit, 2nd Department of Medicine and Laboratory, National and Kapodistrian University of Athens Faculty of Medicine, Athens, Greece

**Keywords:** arthritis, psoriatic, bimekizumab, interleukin-17, treatment outcome, drug-related side effects and adverse reactions

## Abstract

**Background:**

Psoriatic arthritis (PsA) is a multi-domain inflammatory disease affecting skin, nails and the musculoskeletal system, often accompanied by comorbidities that complicate the management of patients. Although several biologic therapies have demonstrated efficacy, real world data for their use in difficult to manage PsA cases is limited. Bimekizumab, a dual interleukin-17A/F inhibitor, offers a promising option in such patients.

**Case presentation:**

We report three challenging PsA cases treated effectively with bimekizumab. The first case involved a woman with isolated axial PsA presenting as inflammatory cervical pain, who achieved remission after intolerance to adalimumab. The second concerned a man with severe psoriasis and morbid obesity, who achieved rapid and sustained remission. The third case refers to a man with refractory PsA, multiple comorbidities and failure of several biologic and targeted therapies. Bimekizumab achieved complete remission of joint and skin manifestations in all three cases.

**Conclusion:**

These real-world cases support the efficacy and tolerability of bimekizumab across diverse and complex PsA presentations. Dual IL-17A/F inhibition may provide a valuable therapeutic option for patients with refractory disease, comorbidities, or rare phenotypes.

## INTRODUCTION

Psoriatic arthritis is a chronic inflammatory disease which affects the skin, nails, axial and peripheral musculoskeletal structures, causing pain, disability and deteriorating the patients’ quality of life.^1^ Moreover, psoriasis and psoriatic arthritis, or -as recently referred to- the psoriatic disease, are associated with an array of comorbidities, such as obesity, metabolic syndrome, cardiovascular disease, fibromyalgia and depression that confer an additional health burden to patients and prompt for multidisciplinary care.^2,3^ Over the past 25 years plenty of new treatments targeting specific inflammatory pathways have become available and more are at the stage of development. All approved compounds have shown their efficacy and safety profile within randomised control trials.^4^ However, special patient populations, such as those with distinct disease manifestations, significant comorbidities or failure to multiple targeted treatments are not specifically investigated or may not be eligible for recruitment in trials at all. Therefore, the efficacy and safety of drugs, particularly the newest ones, in such patients become known through real-world observations. Herein, we report on the efficacy of bimekizumab, a dual interleukin-17 A/F (IL-17A/F) inhibitor in three patients with challenging disease characteristics.

## CASE 1: ISOLATED AXIAL PSORIATIC ARTHRITIS

A 50-year-old woman presented to the rheumatology department with significant deterioration of cervical pain and discomfort over the last months. She reported prolonged early morning stiffness and severe restriction of movement. She was diagnosed with skin psoriasis 8 years earlier on the basis of erythematous plaques with extensive desquamation in the arms and feet. The patient had received topical treatments for psoriasis and methotrexate which was discontinued after 5 months due to gastrointestinal intolerance.

Clinical examination revealed neck pain leading to restriction in head rotation (about 30° left and 40° right) and psoriatic plaques on her arms and head, with a Psoriasis Area and Severity Index (PASI) 11. She did not suffer from peripheral arthritis, enthesitis, dactylitis, low back pain, and she denied symptoms suggesting ocular or gastrointestinal inflammation. Bath Ankylosing Spondylitis Disease Activity Index (BASDAI) at this point was 5.2.

Inflammatory markers were elevated with C-reactive protein (CRP) at 0.63 mg/dl and erythrocyte sedimentation rate (ESR) at 33 mm/h. Rheumatoid factor and anti-CCP antibodies were negative and serum immunoglobulins levels were normal.

**Figure 1.**
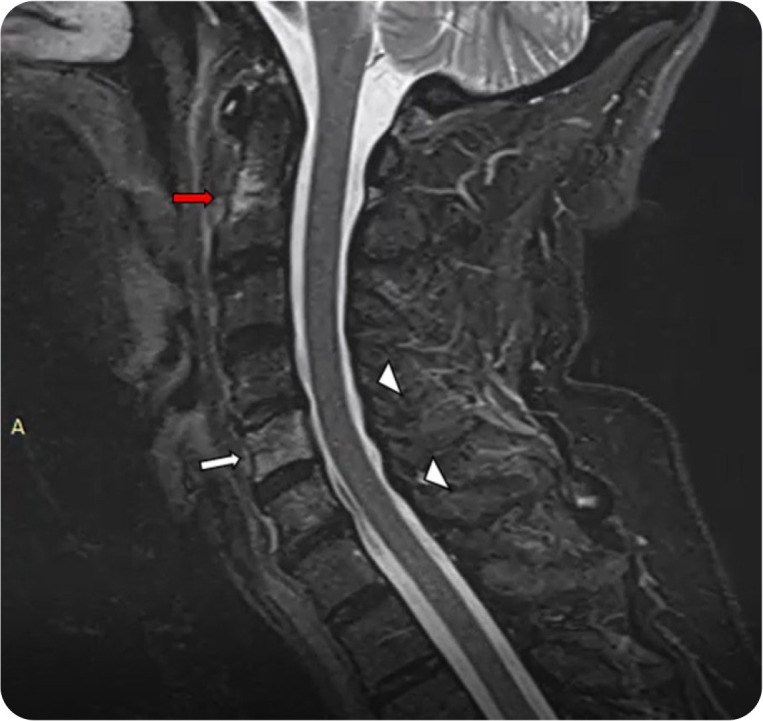
Magnetic resonance imaging STIR sequence: inflammatory spinal lesions- bone marrow oedema in C2 (red arrow) and extensive inflammation in C5 resembling osteitis (white arrow). Degenerative structural changes in C4-C5, C6-C7 (white arrowheads).

**Figure 2.**
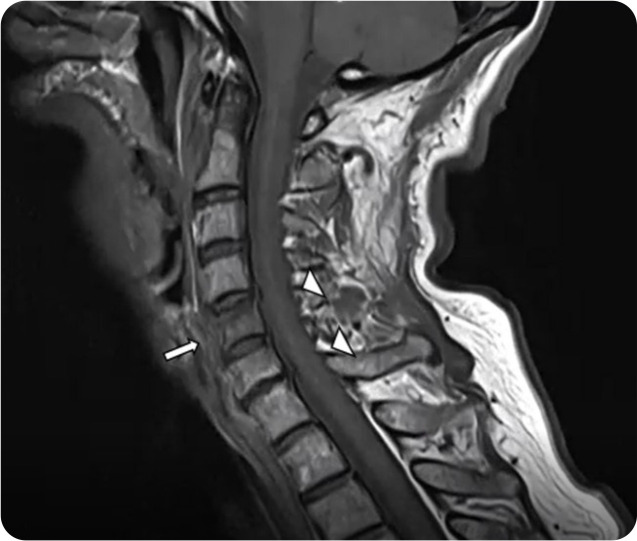
Magnetic resonance imaging T1w sequence: hypointense signal in C5 (white arrow) and degenerative structural changes in C4-C5, C6-C7 (white arrowheads).

The patient underwent magnetic resonance imaging (MRI) of the cervical spine which exhibited bone marrow oedema in C2 and extensive hyperintensity in C5 in STIR sequence resembling osteitis (**Figure 1**). T1 weighted sequences did not reveal fatty lesions but hypointense signal in C5 consistent with active inflammation (**Figure 2**). Degenerative structural changes in C4-C5, C6-C7 were also identified, possibly contributing to mechanical neck problems prior to the change of pain characteristics from mechanical to inflammatory. On the basis of imaging finings, clinical presentation and the history of skin psoriasis the patient was diagnosed with axial psoriatic arthritis.

A 4-week treatment with non-steroidal anti-inflammatory drugs (NSAIDs) provided partial symptom relief, yet CRP remained elevated, and the BASDAI score was 4.1. Thereafter, the patient was started on adalimumab; however, she experienced a severe allergic reaction attributed to the medication, leading to its discontinuation. Subsequently bimekizumab treatment was initiated with good response on both clinical and laboratory grounds, as BASDAI score declined to 2 and inflammatory markers normalised.

Although axial involvement in PsA (AxPsA) was firstly described in 1961, there are still controversies regarding the definition, emphasising the heterogeneity of the disease.^5,6^

Even though the prevalence of axial PsA varies according to population and disease stage, isolated axial involvement has been reported in 2–5% of PsA patients, most of whom show both inflammatory and post- inflammatory changes.^7^ According to the Greek PsA registry, axial PsA was found in 25.8 % of patients, one third of whom presented with isolated spine involvement without sacroiliitis.^8^ It appears that the percentage of isolated spine inflammatory lesions in axial PsA is higher compared to “classic” axial spondyloarthritis, a unique AxPsA feature. The majority of patients with axial PsA present with inflammatory back pain. Cervical pain has been reported in 46.1% of 93 patients with PsA who underwent whole spine MRI.^9^

In our patient, isolated cervical involvement typically presented with inflammatory cervical pain. Dual interleukin 17A/F (IL-17A/F) inhibition has demonstrated efficacy in skin psoriasis, axial spondylarthritis and active PsA in both biologic naïve and experienced patients.^10–13^ According to the Group for Research and Assessment of Psoriasis and Psoriatic Arthritis (GRAPPA) and the European Alliance of Associations for Rheumatology (EULAR) recommendations, IL-17 inhibition has proven efficacy in axial PsA and should be considered as a treatment option, especially when skin disease is active.^14,15^ There is limited data for the use of dual IL-17A/F inhibition in axial involvement in PsA. However, extrapolated data from diseases with similar features suggests bimekizumab is a valid treatment option. Our experience from the case presented here, advocates the use of dual IL-17 A/F inhibition as a therapeutic approach to this special PsA manifestation.

## CASE 2: PSORIATIC ARTHRITIS WITH EXTENSIVE SKIN INVOLVEMENT AND OBESITY

A 52-year-old male patient presented to the combined rheumatology-dermatology clinic with extensive plaque psoriasis for the last 25 years. His medical history was remarkable for morbid obesity with a body mass index (BMI) of 42.3kg/m^2^, arterial hypertension, and dyslipidaemia. The patient had only received topical treatments for psoriasis. Approximately two years before his presentation to our clinic, he had symptoms consistent with peripheral asymmetric oligoarthritis and was also diagnosed with PsA. Oral methotrexate was initiated at a dose of 15 mg per week; however, the treatment was discontinued due to elevated aminotransferase levels.

**Figure 3.**
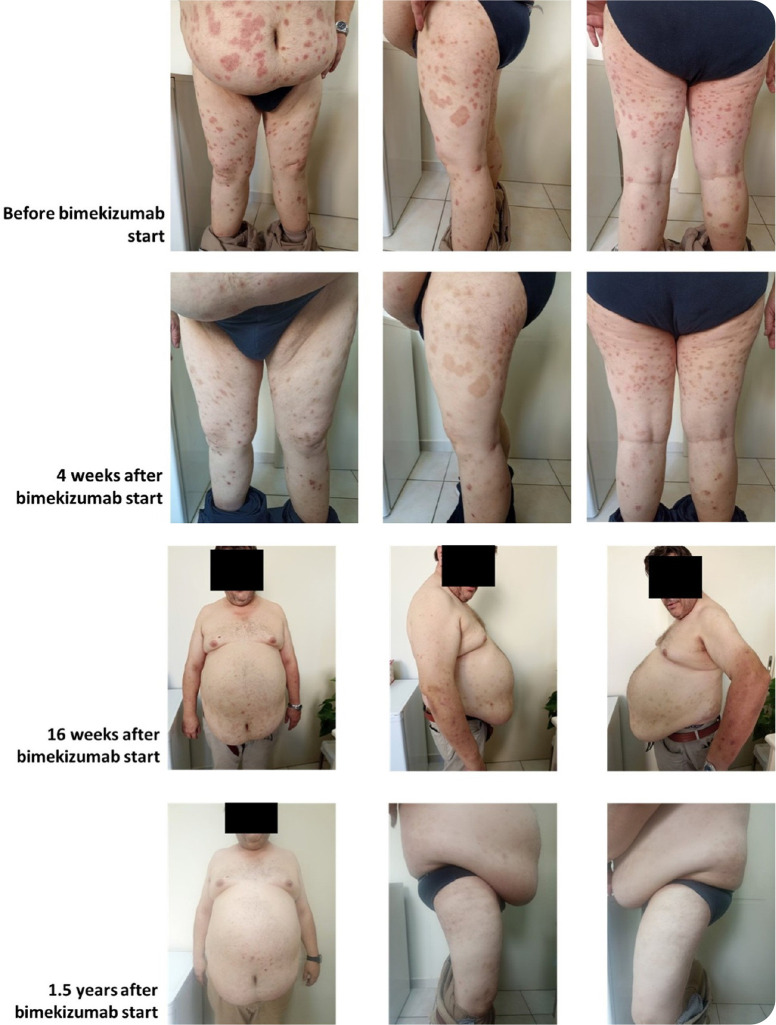
Patient’s plaque psoriasis at baseline, 4 weeks, 16 weeks and 1.5 years after bimekizumab initiation.

On presentation, the patient reported significant impact of his psoriasis on the quality of his life, with moderate pruritus and severe limitations to daily activities due to extensive skin involvement. This was reflected on his Dermatology Life Quality Index (DLQI) which was calculated at 15. The physical examination revealed extensive, thick and scaly plaques covering approximately 18% of Body Surface Area (BSA), while PASI was 11.4 (**Figure 3**). There was also evidence of active peripheral arthritis, with three swollen and four tender joints with a Disease Activity in Psoriatic Arthritis (DAPSA) score of 20.6. Baseline laboratory investigations were notable for elevated CRP of 1.6 mg/dL, but were otherwise unremarkable. Screening for latent tuberculosis and viral hepatitis was negative.

Given the patient’s severe and extensive skin disease, as well as his morbid obesity, which is known to be associated with decreased drug exposure and reduced efficacy of certain biologic therapies, the decision was made to initiate treatment with bimekizumab.^16,17^ The dual interleukin 17A/F inhibition was selected based on the hypothesis that it may provide a more comprehensive inhibition of the IL-17 pathway. This was particularly important in the setting of a high-inflammatory state exacerbated by adiposity. According to bimekizumab approved dosage, PsA patients with moderate to severe psoriasis may receive 320 mg bimekizumab every 4 weeks, continuing beyond 16 weeks in patients ≥120 kg who have not achieved full skin clearance. This flexibility could also benefit our patient with extensive psoriasis and obesity.

Therefore, treatment was initiated with bimekizumab 320 mg subcutaneously every 4 weeks. The patient reported rapid symptom improvement, with a noticeable reduction in scaling and pruritus within the first four weeks. The patient continued to improve and achieved a PASI 90 response by week 16 (**Figure 3**), while at the same time his quality of life improved with a DLQI score falling to 3. There was also significant improvement in his arthritis, with resolution of his peripheral synovitis by week 16, at which point his DAPSA was 6.2.

The treatment was well tolerated throughout the observation period. The patient reported no serious treatment-related adverse events, including no fungal infections and no new or worsening symptoms of inflammatory bowel disease. At his latest follow up, approximately 1.5 years after bimekizumab initiation, the patient maintained an excellent result with both his psoriasis and PsA being in remission and his quality of life appearing significantly improved (DLQI 1).

This case demonstrates that bimekizumab can induce rapid and complete skin clearance in a patient with severe psoriasis and morbid obesity, both being poor prognostic factors for a response to advanced treatments. This robust response suggests that bimekizumab’s dual inhibitory mechanism, as well as the flexibility in dosing may be particularly effective in this challenging patient phenotype. These observations are consistent with real-world data which show that obese psoriasis patients achieve high rates of skin clearance with bimekizumab.^18,19^

## CASE 3: DIFFICULT-TO-MANAGE PSORIATIC ARTHRITIS

A 71-year-old male was referred in November 2021 for evaluation of arthritis of the lower limbs and thick scaly skin lesions on legs that had appeared six months earlier. His medical history included chronic obstructive pulmonary disease (COPD), pulmonary tuberculosis (treated in 2003), chronic hepatitis B virus (HBV) infection, and dyslipidaemia, for which he received atorvastatin and inhaled bronchodilators.

On clinical examination there was arthritis of the ankles and the left knee, large plaques over the shins, on the forehead, scalp and arms, as well as psoriatic nail changes. Laboratory investigations revealed an inflammatory state with normocytic anaemia (haematocrit 30.4%, haemoglobin 10.3g/dL, MCV 97.7fL), leukopenia (leukocytes 3.700/mm^3^, neutrophils 2.150/mm^3^, lymphocytes 590/mm^3^), elevated CRP at 17.83 mg/dL and ESR at 107 mm/h, and mildly elevated liver enzymes (AST 72 U/L, ALT 75 U/L). Aspiration of the left knee and synovial fluid analysis showed 2,400 nucleated cells/μL (80% neutrophils), absence of crystals and negative cultures. The patient was initiated on methylprednisolone, cyclosporine A and entecavir, but soon cyclosporine was replaced with intravenous infliximab due to inadequate response. Although psoriasis and arthritis responded to infliximab, a later flare prompted a switch to ixekizumab. However, the patient’s arthritis showed no response and he was switched back to a tumour necrosis α (TNFα) inhibitor, certolizumab pegol, with only partial response. Following that, he was treated with methotrexate 15mg/week and upadacitinib 15mg/day, resulting in a major skin improvement and remission of arthritis, although mild anaemia, leukopenia, and elevated inflammatory markers persisted.

**Figure 4.**
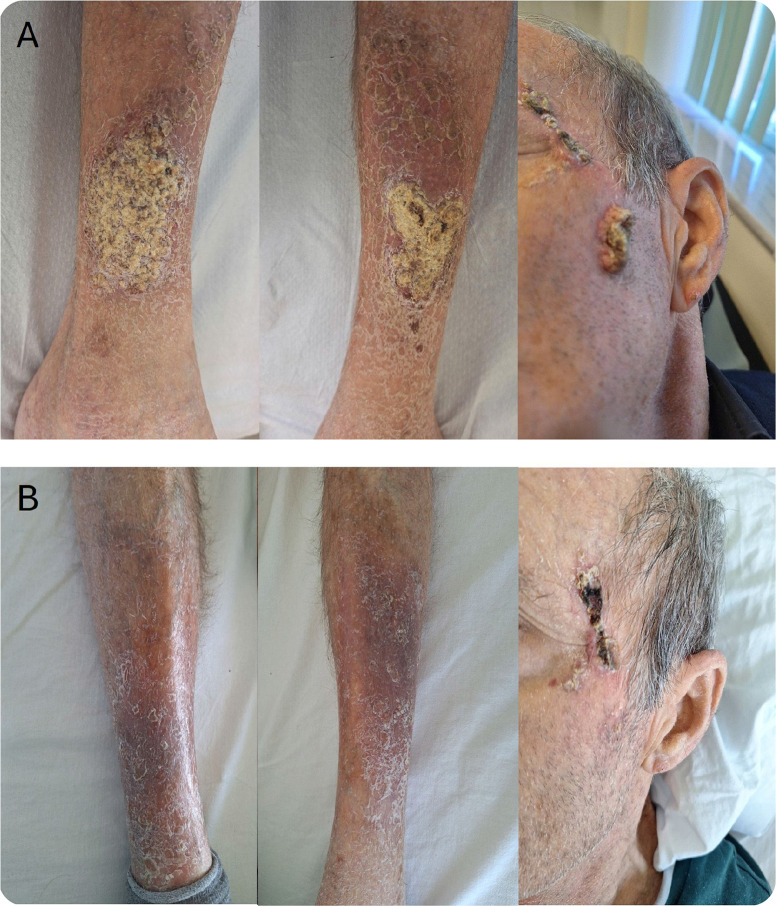
Thick hyperkeratotic psoriatic plaques of the lower extremities and the face before(A) and after 6 months(B) of bimekizumab treatment.

In September 2024, the patient was hospitalised for COVID-19 infection and 2 months later presented with severe anaemia (haematocrit 18.5%, haemoglobin 7.3 g/dL, MCV 122 fL), leukopenia (leukocytes 2.480/mm^3^, neutrophils 2.100/mm^3^, lymphocytes 430/mm^3^) and elevated lactate dehydrogenase (LDH 499 U/L). There was also worsening of the cutaneous lesions, but no active arthritis. The direct Coomb’s test was positive for C3d and cold agglutinins were detected at a titre of 1:32 at 22°C. A workup for cancer and lymphoma was negative and the patient was treated with intravenous pulses of methylprednisolone and rituximab (600 mg weekly for four weeks), with subsequent improvement. However, one month later, a flare of arthritis and a further exacerbation of the skin lesions led to the addition of bimekizumab 160mg every 4 weeks subcutaneously, aiming at the dual blockade of IL-17A and IL-17F. In the next couple of months the patient had complete resolution of arthritis and almost complete clearance of the skin (**Figure 4**). Moreover, during the following 12 months, the cold-agglutinin syndrome remained in remission. Apart from a mild oral candidiasis treated topically, no serious infections occurred despite the close timing of rituximab and bimekizumab initiation.

These difficult-to-treat (D2M) cases often present multiple challenges, such as persistent inflammation, treatment intolerance, comorbidities or secondary complications that limit therapeutic options.^20–22^ As there is no evidence yet on how to manage D2M PsA, available treatment options still comprise a biologic disease-modifying anti-rheumatic drug (bDMARD) targeting TNF, IL-12/23, IL-23, or IL-17 (including a dual IL-17A/F inhibitor), or a targeted synthetic DMARD, such as a Janus kinase (JAK) or a phosphodiesterase-4 (PDE4) inhibitor, in monotherapy or combination with conventional synthetic DMARDs.^23^

The case presented here fits the concept of both D2M and treatment-refractory PsA as recently proposed by EULAR.^24^ Our patient was refractory to multiple lines of treatment, including methotrexate, two TNFα inhibitors, an IL-17A and a JAK inhibitor, while comorbidities, such as chronic HBV infection, a history of tuberculosis and -particularly-cold agglutinin syndrome post COVID-19 for which he had recently been exposed to rituximab, represented safety concerns. As regards safety, IL-17 inhibitors have shown a very low risk of tuberculosis reactivation^25^ and have a good safety profile in patients with chronic HBV infection under anti-viral treatment.^26^ Furthermore, elevated IL-17 levels have been reported in both autoimmune haemolytic anaemia and cold agglutinin disease, although there is no evidence yet to support a direct pathogenic role of IL-17 in these conditions^27,28^ nor a potential effect of IL-17 inhibitors in their treatment.

Concerning refractory PsA, recent reports propose novel strategies, such as biologic combinations to induce remission and targeted synthetic DMARDs to sustain it, although these approaches require careful evaluation of disease activity and comorbidities.^29^ In this context, dual IL-17A/F inhibition represents a reasonable therapeutic approach, offering an alternative mechanism of action with proven efficacy in PsA and a favourable safety profile. Indeed, in our D2M patient bimekizumab led to a sustained response across both skin and joint domains without significant safety issues.

## CONCLUSION

As illustrated in the PsA cases presented above, dual IL-17A/F inhibition with bimekizumab appears efficacious and safe in situations not addressed in clinical trials, such as patients with predominant axial involvement, those with severe psoriasis and concomitant obesity, as well as those with treatment refractory PsA and multiple comorbidities that further complicate their management.
